# Transcriptomics and AI-driven approaches to the diagnosis and treatment of rheumatoid arthritis

**DOI:** 10.3389/fimmu.2026.1812290

**Published:** 2026-04-20

**Authors:** Marzena Ciechomska, Maciej Oldak, Magdalena Massalska

**Affiliations:** 1 Department of Pathophysiology and Immunology, National Institute of Geriatrics, Rheumatology, and Rehabilitation (NIGRiR), Warsaw, Poland; 2 Laboratory of Sequencing, Nencki Institute of Experimental Biology, Warsaw, Poland; 3 Medical University of Lodz, Doctoral School of Molecular Medicine, Lodz, Poland

**Keywords:** algorithms, artificial intelligence, biomarkers, machine learning, precision medicine, rheumatoid arthritis, transcriptomics

## Abstract

Rheumatoid arthritis (RA) is a chronic autoimmune inflammatory disorder marked by joint swelling, pain, and progressive tissue destruction. Increasing evidence suggests that dysregulated RNA expression critically drives RA progression by perturbing immune, inflammatory, and stromal cell programs. These aberrant transcriptional signatures offer valuable biomarkers for diagnosis, prognosis, and therapeutic stratification. Recent advances in transcriptomic technologies have transformed our understanding of RA biology. Bulk RNA profiling has highlighted key dysregulated pathways and disease-associated molecular signatures. Single-cell transcriptomics has expanded this insight by defining extensive cellular heterogeneity and uncovering rare immune and stromal populations implicated in disease initiation, progression, and treatment response. The emergence of spatial transcriptomics provides an additional dimension by preserving tissue architecture, enabling precise localisation of pathogenic cell states and mapping cell–cell interactions within inflamed joints and other affected tissues. Integration of transcriptomic datasets with advanced computational and machine learning (ML) methods has accelerated biomarker discovery. Techniques such as Random Forest, XGBoost, support vector machines (SVM), artificial neural networks (ANNs), and Least Absolute Shrinkage and Selection Operator (LASSO) regression facilitate feature selection and prediction from high-dimensional data. Complementary network- and pathway-based tools, including Weighted Gene Co-expression Network Analysis (WGCNA) and Gene Set Variation Analysis (GSVA), uncover co-regulated modules and refine clinically relevant signatures. Collectively, this review aims to provide an update on how the integration of transcriptomics, spatial technologies, and advanced algorithms offers powerful opportunities to identify novel biomarkers and pathogenic cell populations, thereby advancing precision medicine in RA.

## 1 Introduction

Rheumatoid arthritis (RA) is a chronic autoimmune disease marked by persistent joint inflammation, pain, and progressive tissue damage. Pathogenesis of RA involves a complex interplay of genetic predisposition, environmental triggers, epigenetic modifications, and dysregulated immune signalling (1). Dysregulated immune signalling further reinforces transcriptomic abnormalities. Chronic activation of innate and adaptive immune pathways generates sustained transcription of pro-inflammatory cytokines, chemokines, and matrix-degrading enzymes. Persistent signalling through interferon, TNF, IL-6, and other inflammatory mediators enhances global RNA expression patterns, promoting pathogenic transcriptional modules that maintain immune cell activation and drive tissue damage in RA and other rheumatic diseases (2–6). Moreover, our and other findings indicated that altered RNA signalling can produce disease-specific splicing patterns, aberrant RNA isoforms, and dysregulated expression of microRNA (miRNA) that sustain autoimmunity (3, 6–10).

RNA transcriptomics and spatial transcriptomics, increasingly powered by advanced artificial intelligence (AI) tools, transformed the way RA is classified and managed (11, 12). Bulk RNA sequencing (bulk RNA-seq) and single-cell RNA transcriptomics (scRNA-seq) provide high-resolution insights into the gene expression programs that drive inflammation, autoimmunity, and tissue remodelling (3, 6, 7, 13, 14). By profiling thousands of cells simultaneously, transcriptomic approaches reveal disease-specific molecular signatures, uncover pathogenic cell subsets, and identify dysregulated pathways that traditional immunologic assays cannot detect. Spatial transcriptomics further advances these insights by precisely resolving gene expression within the anatomical architecture of affected tissues, such as the inflamed synovium (15). This technology preserves spatial context that is crucial for RA biology, enabling the mapping of immune–stromal interactions, the identification of microanatomical inflammatory niches, and the characterisation of gradients of cytokine signalling or cellular activation that contribute to chronic inflammation and joint tissue destruction (16, 17). Furthermore, RA is more heterogeneous than many other diseases, with substantial variability in clinical and molecular features (18). Therefore, approaches such as bulk RNA-seq, scRNA-seq and spatial transcriptomics are needed to capture this complexity. Integrating these data with an AI-based approach enables a deeper understanding of disease mechanisms and supports improved therapeutic strategies.

Machine learning (ML) methods, a core component of AI, are now essential for interpreting the vast and complex datasets generated by these technologies (19). ML models can integrate multi-modal data combining gene expression, spatial localisation, histopathology, and clinical phenotypes in order to define molecular subtypes of disease, predict therapeutic responses, and identify novel biomarkers. Deep-learning algorithms enhance cell-type classification, detect rare pathogenic populations, and reveal emergent patterns in tissue organisation that are not apparent through manual analysis. In spatial transcriptomics, AI further supports the reconstruction of cellular neighbourhoods, quantifies crosstalk between immune and stromal cells, and delineates tissue microenvironments that correlate with disease severity or progression.

**Figure 1:**
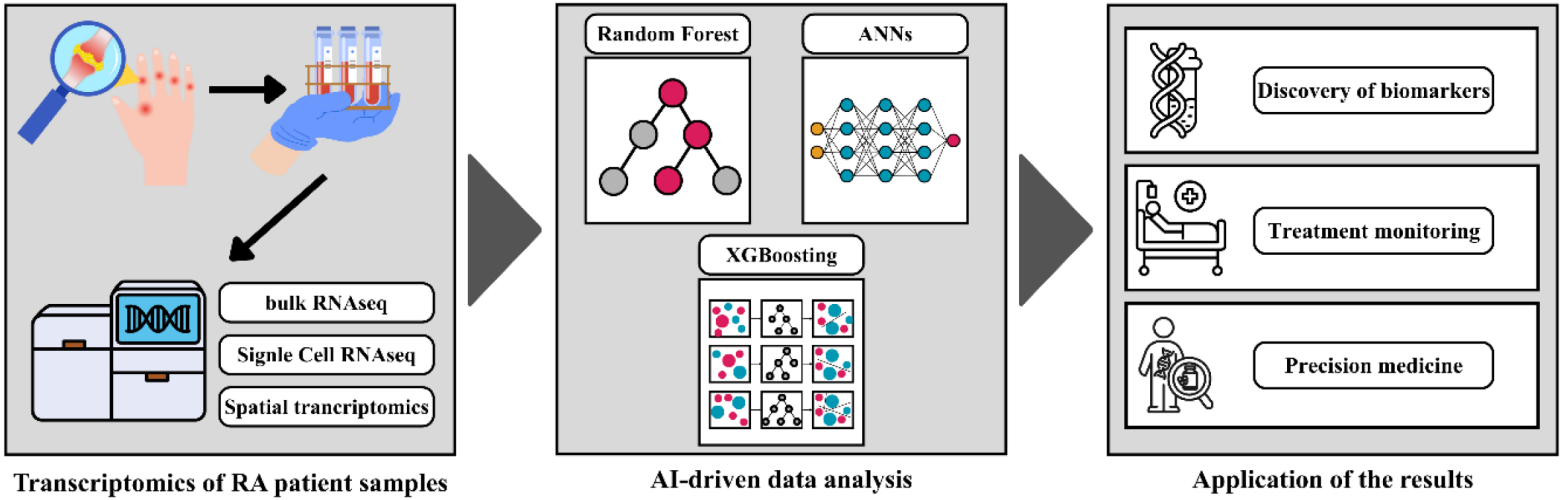
Workflow of the RA patient samples processing integrating bulk-RNA sequencing, single cell RNA-sequencing and spatial transcriptomics with AI-driven analytics in order to enhance the precision of disease diagnosis and the monitoring of therapeutic responses.

Together, RNA transcriptomics, spatial transcriptomics, and AI-driven analytics provide an integrated framework that moves rheumatology towards a molecularly informed discipline. These tools illuminate the dynamic interplay between immune dysregulation, tissue-specific responses, and microenvironmental factors. They also lay the foundation for precision medicine approaches by identifying targeted pathways for intervention, refining patient stratification, and informing therapeutic decision-making. Ultimately, these technologies are reshaping our understanding of RA by revealing its complexity at cellular and spatial resolution, advancing both mechanistic insight and clinical application. An overview of the workflow for processing RA patient samples, integrating bulk RNA sequencing, single-cell RNA sequencing, and spatial transcriptomics with AI-driven analytical frameworks to improve diagnostic precision and therapeutic response monitoring can be seen in **Figure 1**.

In this review, we synthesise recent advances and limitations in RNA transcriptomics, spatial transcriptomics, and AI-driven approaches that markedly enhance the precision of disease diagnosis and the monitoring of therapeutic responses in RA.

## 2 Comparative ML models used in RA

The expanding availability of transcriptomic and clinical datasets in RA has generated a need for advanced analytical frameworks capable of extracting biologically and clinically meaningful insights from high-dimensional data. AI and ML approaches have consequently become central to transcriptomic research, enabling improved disease classification, biomarker discovery, and prediction of therapeutic responses.

Medical data analysis relies on algorithms that detect subtle patterns, reduce dimensionality, and achieve high predictive accuracy. In rheumatology, supervised learning models such as Random Forest and Extreme Gradient Boosting (XGBoost) are widely applied for patient stratification and outcome prediction due to their robustness and ability to handle complex, nonlinear relationships. Feature selection and network inference techniques, including the Least Absolute Shrinkage and Selection Operator (LASSO) and Graphical LASSO (GLASSO), facilitate the identification of sparse gene signatures and regulatory interactions underlying disease mechanisms. Systems-level approaches such as Weighted Gene Co-expression Network Analysis (WGCNA) enable the detection of co-expressed gene modules associated with clinical traits, whilst pathway-based methods like Gene Set Variation Analysis (GSVA) support functional interpretation of transcriptomic profiles. Model performance is commonly evaluated using metrics such as the Matthews Correlation Coefficient (MCC), which is particularly informative for imbalanced datasets frequently encountered in rheumatic disease studies. The principles, applications, and comparative advantages of Random Forest, XGBoost, LASSO, GLASSO, WGCNA, and MCC will be further explained in the following sections.

### 2.1 Random Forest

**Figure 2:**
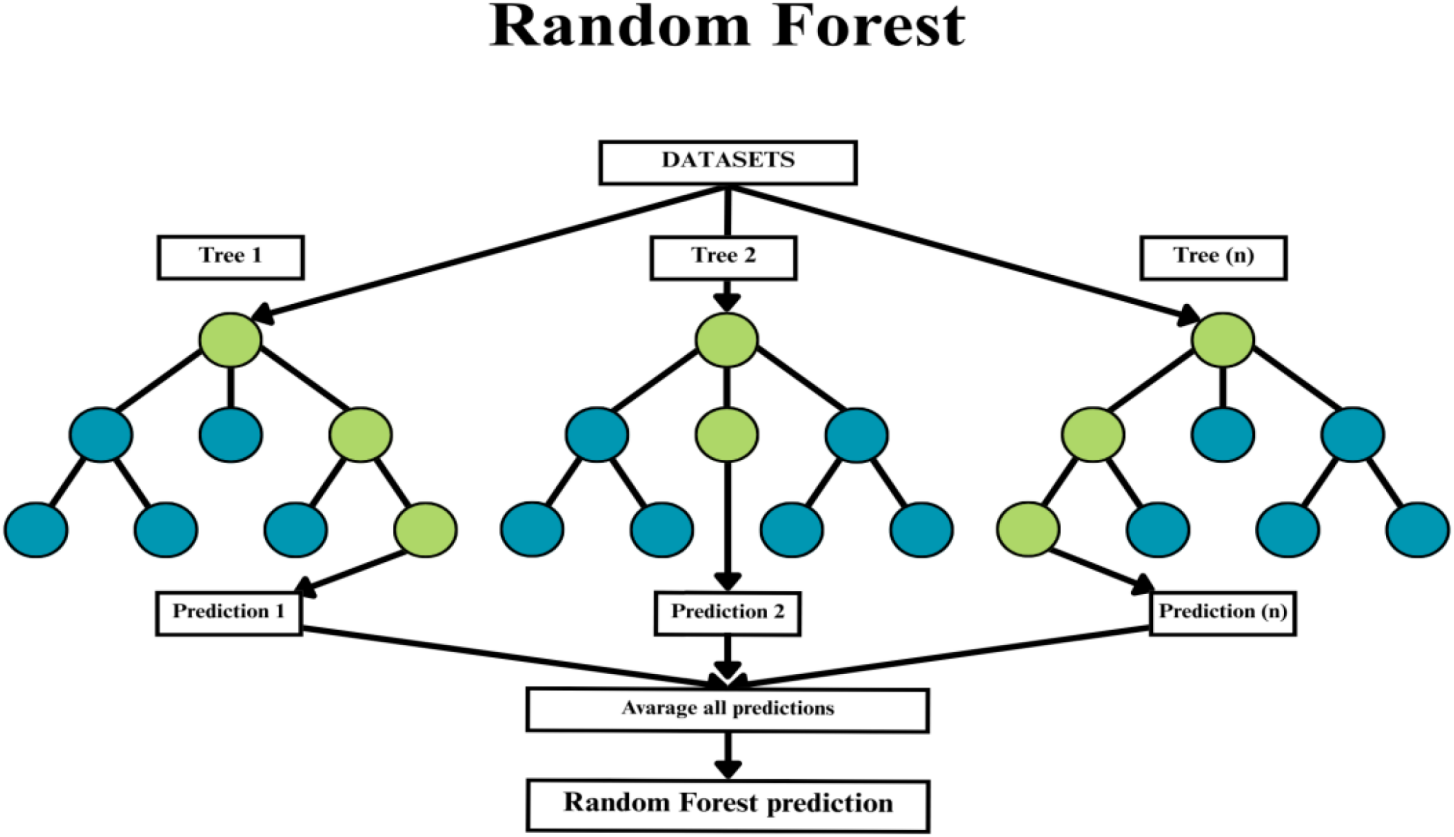
Illustration of random forest operation tree diagram. It is an ensemble learning algorithm that builds multiple decision trees and aggregates their predictions to improve classification accuracy and reduce overfitting. Circles represent single answers in the Random Forest Tree. Bright green pathway is the way from input data to the result. Another colour (here it is turquoise) represent the possible answer from the single Random Forest Tree.

Random Forest is a classification and regression algorithm belonging to the group of ensemble methods (20). It creates multiple decision trees, each of which is trained on random data and variables (**Figure 2**). The result is an average (for regression) or majority vote (for classification) of the results of individual trees (20, 21). Random forest is a method that is resistant to overfitting and performs well with data that has a large number of features (21). For this reason, this method is often used in gene expression analysis, the diagnosis of diseases or clinical sample classification (22). What is more, Random Forest provides important indicators, which allow to define the most important biological variables (22, 23).

### 2.2 Weighted gene co-expression network analysis

WGCNA is a method of gene co-expression analysis that enables the identification of gene modules with similar expression patterns (23, 24). In that method, genes are treated as network nodes, and the edges represent the strength of correlation between their expression (24, 25). WGCNA allows linking gene modules to phenotypic characteristics such as tissue type or disease stage (24). Thus, the biological interpretation can be facilitated. This method is often used as a first step of predictive analysis. Identified gene modules may be a subject for further analysis using LASSO or Random Forest to indicate biomarkers of greatest diagnostic significance (24, 25).

### 2.3 Genetic set variation analysis

**Figure 3:**
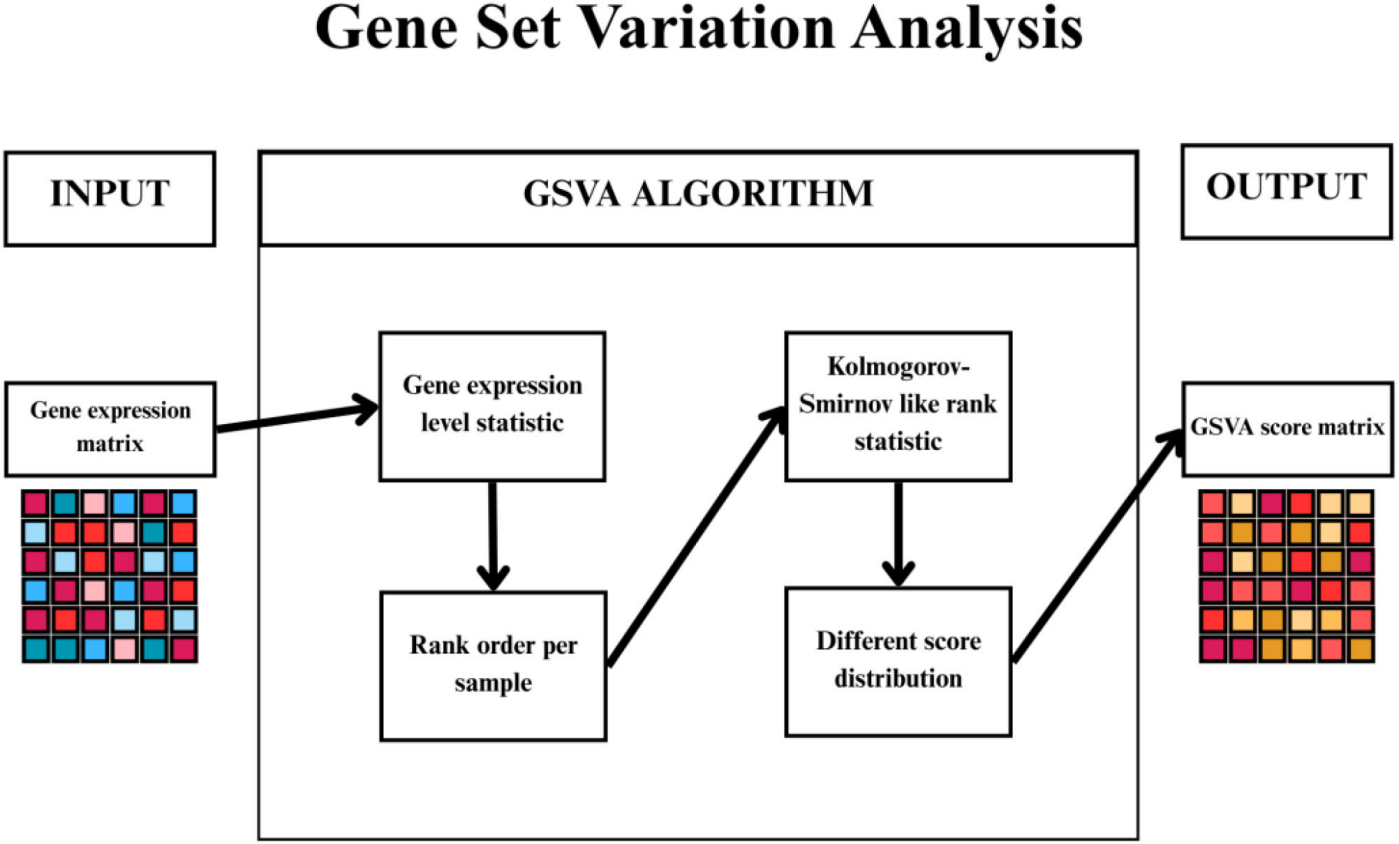
Illustration of the operational diagram of gene set variation analysis operation. gene set variation analysis (GSVA) estimates pathway activity across samples by converting gene expression data into gene set–level enrichment scores, enabling pathway-level interpretation of transcriptomic profiles.

GSVA is based on algorithms inspired by evolutionary processes for variable selection. The population of possible solutions evolved through crossover, mutation and selection operators to maximise a specific objective function (26, 27). The advantage of GSVA is the ability to explore large feature spaces and identify non-linear relationships. In bioinformatics, GSVA is often used to select the most important genes, or variables of greatest biological significance (**Figure 3**) (26, 28).

### 2.4 Extreme gradient boosting

**Figure 4:**
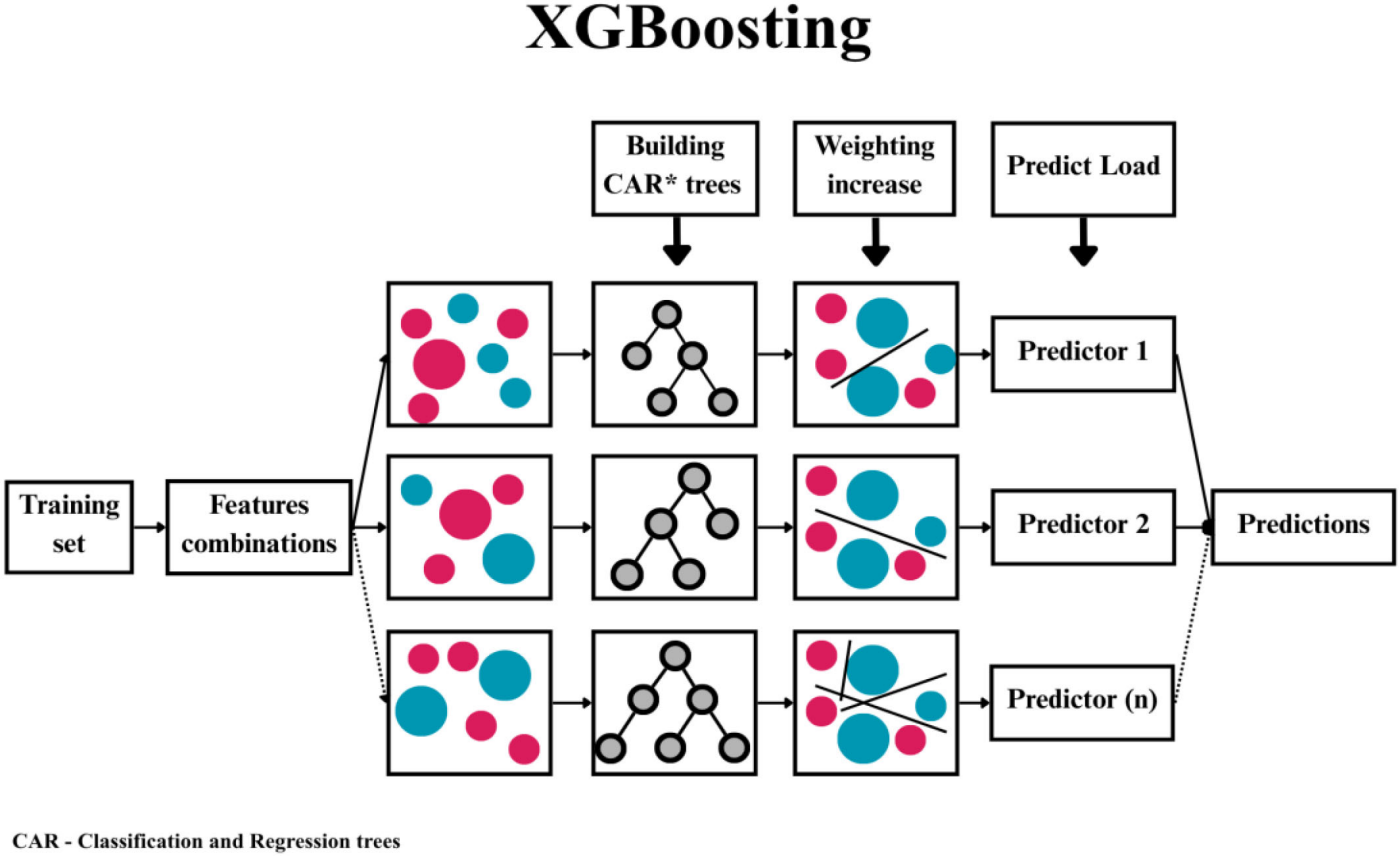
Architecture of the XGBoost model. The algorithm constructs feature sets and, based on them, analyses weighted outputs from decision trees to determine the final group predictor. Different colours and sizes represent different input data.

XGBoost is an extension of the gradient boosting algorithm and is one of the most effective tools in ML (**Figure 4**). It works iteratively, building successive decision trees that correct the errors of previous ones (29). This tool is characterised by high-performance regularisation capabilities and resistance to incomplete data. It is often used in clinical and omics data, which present higher accuracy than traditional statistical models (29, 30).

### 2.5 Matthew correlation coefficient

**Figure 5:**
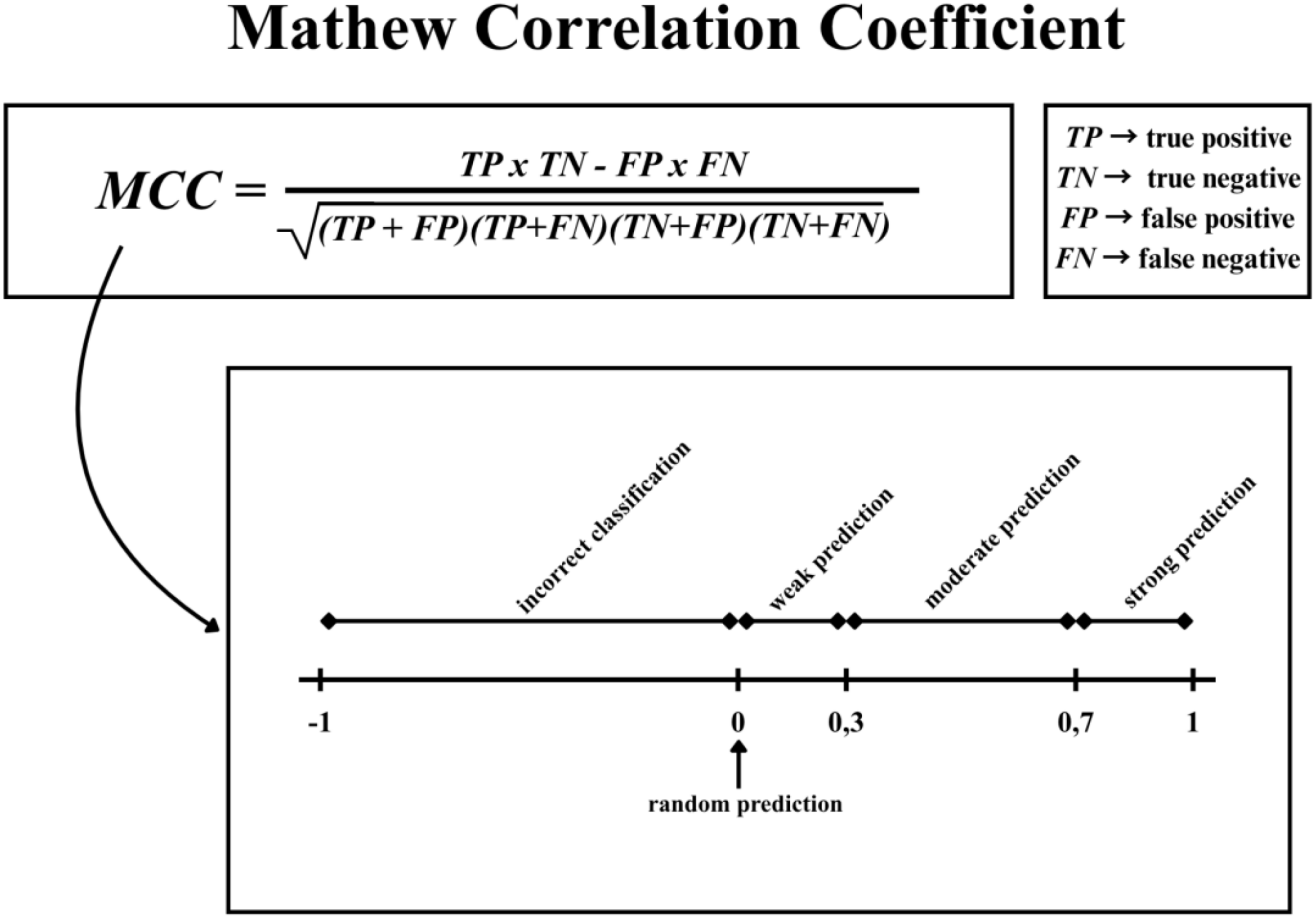
Scheme for calculating the matthews correlation coefficient (MCC). The principle of using true positive (TP), true negative (TN), false positive (FP), and false negative (FN) results, as well as the method for interpreting prediction strength depending on the correlation value.

MCC is a metric for evaluating the quality of binary classification, especially important for unbalanced data sets. As it seen on **Figure 5**, it considers all four elements of the confusion matrix (true positives, true negatives, false positives and false negatives), resulting in higher accuracy (31). MCC values range from -1 to 1, where 1 is a perfect match, 0 is a random result, and -1 is incorrect classification. In biomedical research, where some classes are often less numerous, MCC can provide a reliable assessment (31, 32).

### 2.6 Least Absolute Shrinkage and Selection Operator and Graphical LASSO

LASSO is a linear regression technique using L1 regularisation, which involves adding a penalty proportional to the absolute value of the regression coefficients to the objective function (12, 33). The penalty allows us to identify the most important variables and limit the influence of less significant ones (33).

GLASSO extends this concept by adding L1 penalty to the elements of the inverse covariance matrix. Thus, due to this tool it is possible to obtain a network structure of dependencies between variables, where non-zero values show direct relationships (34). To summarise, LASSO focuses on the selection of predictors in regression models, whilst GLASSO reveals the structure of relationships between variables (33, 34).

### 2.7 Support Vector Machine - Recursive Feature Elimination

SVM-RFE is a method based on the Support Vector Machine (SVM) classifier, a classifier mainly used in high-dimensional data analysis (28). The model’s operation is based on its training iteratively and evaluating the importance of each feature based on the weights assigned by the model. Features with the least significance are gradually removed from the dataset. The process is repeated to obtain the most informative subset of variables (28, 29).

### 2.8 Artificial neural networks

**Figure 6:**
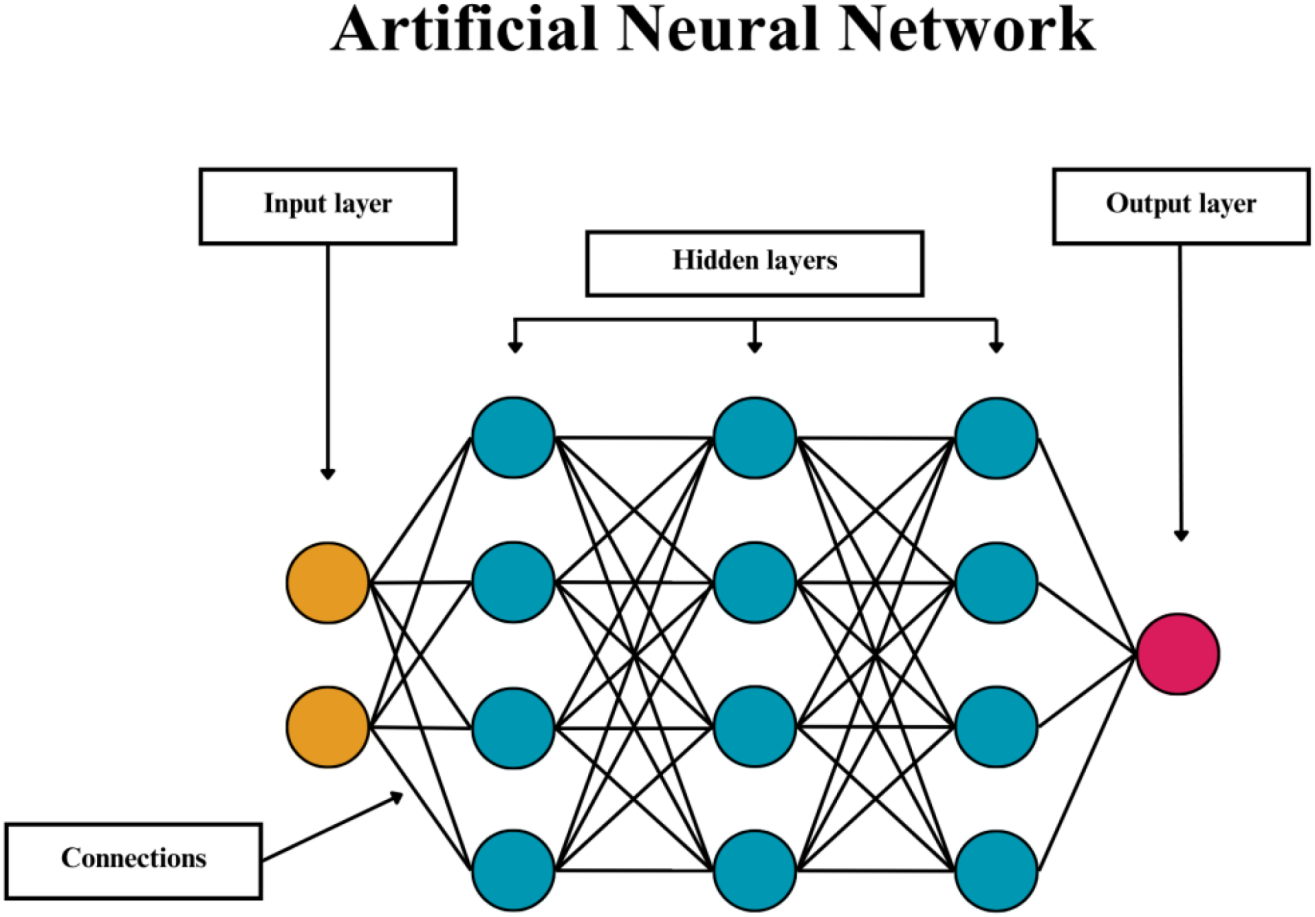
Illustration of artificial neural network operation tree diagram. Neural networks assign weights to each input, determining their relative importance, and then process this information through successive layers, where nonlinear activation functions enable the learning of complex relationships between input data and the model output. Circles are decisions nods. Yellow colour represents input data, turquoise – individual intermediate results in hidden layers, red – obtained results.

ANNs are statistical models inspired by the structure and functionality of biological neurons, which mimic the brain’s learning dynamics (30). Various definitions describe this type of model; however, in general, ANNs refer to neural networks used for non-linear statistical analysis. Inspired by the hierarchical organisation of neurons and synaptic connections, ANNs assign weights to each input, determining their relative importance, and activate when the weighted sum exceeds a defined threshold - the operating diagram is shown in **Figure 6** (31). ANN’s network consists of at least three layers of interconnected units (perceptrons): an input layer that receives data (related to the research question), at least one hidden layer that processes and transforms the information and an output layer that generates the prediction or classification. Their architecture allows ANNs to model complex multi-dimensional relationships in data, making them powerful tools in data modelling and analysis (31, 32).

Listed algorithms present different approaches to data analysis. Random Forest and XGBoost are powerful predictive models, whilst GAVA, SVM-RFE, and LASOO support feature selection. GLASSO and WGCNA assist in understanding the structure of biological interdependencies, and MCC provides a solid evaluation of classification models. On the other hand, ANN bridges the gap between classic ML and modern deep learning techniques. A proper combination of these techniques enables us to create nonlinear models that describe complex biological processes.

## 3 ML for RNA-based biomarker discovery in RA

RA is a clinically heterogeneous and biologically complex disease characterised by substantial molecular diversity, which limits the robustness and reproducibility of conventional biomarker discovery approaches. In this context, bulk RNA-seq provides a comprehensive view of transcriptomic alterations across patient populations, and when combined with ML approaches, enables the identification of complex, multigene expression patterns, thereby enhancing biomarker discovery and patient stratification in RA.

ML, which is a subset of AI, has emerged as a powerful approach for biomarker discovery, particularly when applied to large-scale transcriptomic datasets in RA. Transcriptomic datasets relevant to this type of analysis typically include whole-cell RNA (mRNA), miRNA, as well as other non-coding RNA such as circular RNA. By uncovering complex, non-linear patterns within high-dimensional gene expression data, ML enables the identification of robust molecular signatures associated with disease progression and therapeutic response. Here, we present recent examples of the practical application of ML in biomarker discovery for RA, providing a robust framework for elucidating molecular mechanisms, improving patient stratification, and facilitating the development of precision medicine strategies in RA (**Table 1**).

**Table 1:** Candidate biomarkers identified in RA from transcriptomic studies, highlighting the GEO datasets analysed, and the computational algorithms applied.

Biomarkers discovery in RA from transcriptomic studies			
GEO dataset	Algorithm	Biomarker	Ref.
	Random forest, GLASSO	MZB1	(35)
	SVM-RFE, ANN, LASSO, WGCNA	MMP9, CXCL10, IL15, FOXO3	(36)
GSE45291	WGCNA	UHMK1, APOC2, SFT2D1, ELK4	(37)
GSE77298	GSVA, WGCNA, LASSO, SVM-RFE, XGBoost, Random Forest, ANN	TMEM45A, ZBTB25	(38)
GSE89408 GSE55235	LASSO SVM-RFE	S100A8, ABCC4, VAMP2, PIM2, ISG20	(40)
GSE55457 GSE55584 GSE55235 GSE12021	MCC SVM-RFE	STAT1, JUN, MYC, EGFR	(41)
	GSVA	IL1B, S100A8, NFKB1, STAT3	(42)
GSE12021 GSE55235 GSE55457 GSE77298 GSE89408	LASSO Random Forest	GPR65, STAT1, PTTG1, C1QA, SHTN1, FCGR3B, TMEM176B, CD74, SLC7A7	(43)
	LASSO	hsa-circ0001946 hsa-circ0002715 hsa-circ0000836	(46)

Graphical least absolute shrinkage and selection operator (GLASSO), support vector machine - recursive feature elimination (SVM-RFE), artificial neural networks (ANN), least absolute shrinkage and selection operator (LASSO), weighted gene co-expression network analysis (WGCNA), genetic set variation analysis (GSVA), extreme gradient boosting (XGBoost), matthews correlation coefficient (MCC).

Based on a cohort of 97 RA patients, Yap et al. applied Random Forest classifier models to identify biomarkers predictive of adalimumab treatment response at 6 months using whole-blood transcriptomics (35). The Random Forest classifiers, combined with hyperparameter optimisation, identified the top 30 differentially expressed genes (DEGs) with high predictive accuracy, achieving an area under the curve (AUC) of up to 0.86. Furthermore, implementation of the GLASSO network method, which visualises DEGs interactions and provides biological contextualisation, revealed Marginal zone B and B1 cell-specific protein (MZB1) as a central hub in both responder and non-responder groups. Given that MZB1 plays a role in B-cell development and antibody production, and elevated expression is associated with an approximately 50% higher likelihood of developing anti-drug antibodies, reduced MZB1 expression may mechanistically explain the more favourable treatment response observed in RA patients.

Another study investigated the therapeutic potential of taurine in RA through the analysis of transcriptomic datasets (36). Following the identification of DEGs, SVM-RFE was applied to select the most predictive genes from the complex genetic data, enhancing the precision of the initial screening. To further validate these findings, an ANN model was employed to uncover complex non-linear relationships within the high-dimensional dataset, enabling the identification of key genetic features potentially overlooked by traditional linear approaches. Subsequently, LASSO regression analysis and WGCNA were conducted to construct an optimised predictive model, which highlighted MMP9, CXCL10, IL15, and FOXO3 as significant biomarkers associated with RA, cellular senescence, and autophagy. Additionally, molecular docking and 3D structural analyses revealed that FOXO3 has the potential to bind and interact with the anti-inflammatory molecule taurine, supporting its relevance in therapeutic development.

In RA patients with inadequate responses (IR) to both DMARDs and TNF inhibitors, WGCNA of the GSE45291 dataset identified 17 hub genes strongly associated with RA pathogenesis (37). Among these, four genes—UHMK1, APOC2, SFT2D1, and ELK4—were further validated in a collagen-induced arthritis (CIA) rat model and in LPS-stimulated RAW264.7 cells. Consistent with the patient dataset, expression of these four genes was significantly reduced in both *in vivo* and *in vitro* models, suggesting their potential involvement in inflammatory processes underlying RA. Nevertheless, additional functional studies employing gene knockout and overexpression approaches are required to confirm the mechanistic roles of these candidate genes.

In other analyses, Chen et al. were able to identify TMEM45A and ZBTB25 as mitophagy-related biomarkers (38). Mitophagy, a specialised form of autophagy based on the selective removal and degradation of mitochondria, also plays a critical role in the progression of RA (39). Indeed, by integrating GSVA and WGCNA analysis from the GSE77298 dataset, 2191 core module genes were selected (38). Subsequently, by applying four different ML algorithms — LASSO, SVM-RFE, XGBoost, and Random Forest — 9, 6, 6, and 39 genetic biomarkers were identified, respectively. Further analysis using Receiver Operating Characteristic (ROC) curves showed that TMEM45A had an AUC of 0.991 and ZBTB25 an AUC of 0.911. Also, the disease classification model developed through ANN confirmed high diagnostic accuracy of TMEM45A and ZBTB25 as mitophagy-related biomarkers in RA patients.

In another study by using LASSO and SVM-RFE algorithms, the authors were able to determine the DEGs as telomere-related biomarkers in RA by analysing transcriptome data from a GSE89408 dataset (150 RA tissue samples and 28 control tissue samples) as a training set and GSE55235 (10 RA tissue samples and 10 control tissue samples) as a validation set (40). Subsequently, a nomogram model was constructed in order to select the top 5 candidates, including S100A8, ABCC4, VAMP2, PIM2, and ISG20. The model showed outstanding predictive accuracy for RA, with an AUC of 0.999 and a calibration curve that closely matched the ideal curve, confirming excellent diagnostic performance. Additionally, the nomogram provided greater net clinical benefit than any single biomarker, demonstrating its superior clinical utility. The binding energy analysis revealed strong binding affinity between ABCC4, S100A8, and ISG20 with methotrexate (first-line drug for RA), suggesting that these biomarkers identified through integrated bioinformatic and ML analyses could be potential targets for the treatment of RA.

Using three datasets (GSE55457, GSE55584, and GSE55235), comprising 20 healthy control (HC) and RA samples as the training set, and GSE12021, containing 10 HC and 12 RA samples as the validation set, Ji et al. applied MCC algorithm, along with three ML screenings including LASSO regression, random forest, and support vector machine recursive feature elimination (SVM-RFE) to identify specific genes associated with aging in RA (41). This study successfully identified four biomarkers related to ageing in RA, which were subsequently confirmed by ROC analysis, reaching AUC value of 0.94 for STAT1, 0.87 for JUN, 0.88 for MYC and 0.91 for EGFR.

GSVA of bulk RNA-seq data was also performed as a validation cohort, comprising samples from 19 HC and 8 RA patients. This cohort was compared to an exploratory cohort of 8 RA samples that had been previously validated using scRNA-seq. The analysis revealed that IL1B, S100A8, NFKB1, STAT3 were significantly upregulated in the neutrophil-positive RA group (42). Additionally, five datasets (GSE12021, GSE55235, GSE55457, GSE77298, and GSE89408), comprising a total of 213 RA and 63 HC samples, were integrated to analyse 4–232 commonly detected gene symbols. Subsequently, LASSO regression was applied, followed by Random Forest analysis. This approach consistently prioritised GPR65, STAT1, PTTG1, C1QA, SHTN1, FCGR3B, TMEM176B, CD74, and SLC7A7 as key features (43). Moreover, functional and animal experiments demonstrated that STAT1 activation upregulated synovial LC3 and ACSL4, suggesting that STAT1 may contribute to RA pathogenesis by modulating autophagy and ferroptosis pathways.

Circular RNA (circRNA) can also serve as a biomarker in RA blood samples (44, 45). Using ROC analysis and the LASSO model combined with clinical characteristics of biomarkers for RA diagnostic modelling, three circRNAs were determined. Indeed, with AUC values at 0.75 for hsa-circ0001946, 0.87 for hsa-circ0002715, and 0.66 for hsa-circ0000836, which were better than the classical RA marker rheumatoid factor, reaching only AUC at 0.51 (46). Subsequently, qPCR analysis of plasma exosomes in TNFi patients found that the expression of hsa-circ0002715 was higher than that in patients who didn’t reach the American College of Rheumatology 20% improvement criteria (ACR20). In contrast, the expression of hsa-circ0001946 was significantly lower than that in patients who didn’t reach ACR20. These studies indicate that elevated expression of hsa-circ0002715 and reduced expression of hsa-circ0001946 may serve as potential biomarkers for TNFi precision therapy in RA patients. Overall, these recent examples clearly show that combining high-throughput transcriptomic analysis with ML can identify novel molecular signatures underlying RA pathogenesis. This approach can also improve patient stratification by distinguishing responders and non-responders to specific therapies. Furthermore, it can accelerate the discovery of predictive biomarkers that guide personalised treatment strategies.

## 4 ML in single-cell and spatial transcriptomics in RA

RA arises from a complex interplay of genetic, hormonal, and environmental factors that drive dysregulated immune responses and autoimmunity, resulting in substantial interpatient heterogeneity at both the clinical and molecular levels. This complexity makes RA particularly well-suited to advanced, data-driven approaches. In this context, scRNA-seq and spatial transcriptomics provide complementary insights by enabling high-resolution characterisation of cellular heterogeneity and the spatial organisation of inflamed synovial tissues. Furthermore, integration of these high-dimensional datasets with AI-based analytical tools provides a powerful framework for dissecting pathogenic mechanisms. Ultimately, such approaches may support the validation of existing therapeutic strategies and facilitate the identification of novel, more precisely targeted interventions. In particular, scRNA-seq is a technology that enables the study of gene expression at the level of individual cells. Unlike classical or bulk RNA-seq, which analyses the average gene expression from thousands of cells, scRNA-seq allows for the precise determination of which genes are active in each specific cell, secondly, how cells differ within the same sample, and finally, what types of cells are present in a given tissue or disease.

The scRNA-seq analysis process consists of several main steps. The first is the isolation of single cells (e.g., using microwells, droplets, or micropipettes). Next comes the extraction and reverse transcription of RNA into cDNA. The subsequent step is sequencing the cDNA, followed by bioinformatics analysis, where AI plays an increasingly important role. Data from scRNA-seq experiments are very complex and noisy, containing millions of reads, covering hundreds of thousands of cells and thousands of genes. For this reason, AI algorithms, particularly ML and deep learning, prove indispensable at many stages of analysis.

One of the first steps in data analysis is dimensionality reduction and visualisation, because scRNA-seq data are very high-dimensional (up to 20–000 genes per cell). The most used methods include PCA, t-SNE, and UMAP — classical approaches that enable data visualisation in two or three dimensions. Autoencoders, which are neural networks that learn simplified data representations, are also increasingly used. An example is single-cell variational inference (scVI) — a deep learning model designed to model scRNA-seq data. The next step is cell clustering, i.e., grouping cells with similar gene expression profiles. Both classical clustering algorithms such as K-means, hierarchical clustering, and DBSCAN, as well as more advanced graph-based methods like Louvain or Leiden, are used for this purpose. Deep models such as the deep count autoencoder (DCA), which handle non-standard data distributions better, are also becoming more popular. An important element of scRNA-seq analysis is automatic cell type annotation. AI can recognise cell types based on gene expression patterns using supervised classifiers such as SVM, Random Forest, or neural networks. Transfer learning techniques are also applied, where models are trained on large reference datasets (e.g., the Human Cell Atlas) and then adapted to specific cases.

AI also supports the analysis of cell trajectories (lineage inference), which attempts to reconstruct the differentiation paths of cells (e.g., from stem cells to specialised cells). Methods such as Monocle, Slingshot, or PAGA are used for this purpose. Deep learning models and graph neural networks are particularly effective in modelling complex, branched differentiation pathways. Another important application of AI in scRNA-seq analysis is the integration of multiple datasets, e.g., from different experiments, technologies, or patients. Models such as Scanorama, Harmony, scVI, or Seurat (in versions extended with ML methods) enable efficient data merging, batch effect removal, and improvement of result quality.

The development of scRNA-seq and spatial transcriptomics technologies has revolutionised the way researchers analyse complex tissues, enabling simultaneous capture of gene expression at the single-cell level and their spatial distribution within tissue structures. The spatial analysis of tissue is a unique technique that enables the investigation of cells within their natural anatomical context. This approach preserves information about cell localisation, allowing for a deeper understanding of intercellular communication processes as well as the exploration of tissue heterogeneity. In particular, in the context of inflammatory and autoimmune diseases such as RA, these technologies allow for the identification of unique RNA signatures assigned to specific cell types in synovial tissue and a better understanding of the disease architecture of the joint microenvironment.

There is slowly growing evidence pointing to the published results in field of rheumatology based on scRNA-seq with the significant support of AI, with a few examples presented below and in **Table 2**. Seven years ago, three different synovial subtypes were described by ML, combining gene expression with histological data: a highly inflammatory one (with extensive infiltration of leukocytes), a low inflammatory subtype (with higher expression of neuronal genes, glycoproteins and transforming growth factor β pathway) and a mixed subtype (47).

**Table 2:** Cell phenotypes discovery in RA by using ML.

Cell phenotypes discovery in RA by using ML			
Dataset	Algorithm	Phenotype	Ref.
mass cytometry clustering, transcriptomics	tSNE	*CD90^+^CD34^-^HLA-DRA^hi^* *IL-1β^+^* monocytes, *ITGAX^+^TBX21^+^* B cells *PDCD1^+^* T cells	(48)
scRNA-seq	Monocle	*PRG4^+^* and *POSTN^+^* SFs correlated with disease parameters in fibroid and lymphoid pathotypes respectively, CXCL14^+^SF showed negative correlation with disease severity in all pathotypes	(49)
eQTL, GWAS, scRNA-seq and bulk RNA-seq	XGBoost, GBM, NNET, SVM, KNN	ICOS^+^, IL6ST^+^ and PPP1CB^+^ T cells as risk factors for RA; GADD45A^+^, CD3D^+^, SLFN5^+^, PIP4K2A^+^ and MIER1^+^ T cells as protective factors for RA	(51)
spatial transcriptomics	Deep topic	CD34^+^ fibroblast-like synoviocytes and LYVE1^+^ MERTK^+^ macrophages	(52)
sorted-cell subsets and scRNA-seq	Graph-based Gene expression Module Identification (GbGMI)	Netrin-4^+^ SF augments branching of pain-sensitive CGRP^+^ sensory nerves	(53)

Combining mass cytometry clustering (supported by tSNE algorithm) and transcriptomics, Zhang et al. managed to reveal phenotypes of cells expanded in RA synovia: *CD90^+^CD34^-^HLA-DRA^hi^* sublining fibroblasts, being major source of IL-6, *IL-1β^+^* pro-inflammatory monocytes, *ITGAX^+^TBX21^+^* autoimmune-associated B cells and *PDCD1^+^* T peripheral helper and follicular T cells (48). A deeper understanding of the RA pathogenesis was achieved by an association done between distinct synovial pathologies and synovial fibroblasts (SF) subtypes defined by scRNA-seq, supported by advanced ML methods used to find transcriptomic changes (49). RA SFs have been divided into four groups: *PRG4^+^* SF (lining layer), *CXCL12^+^* SFs, *POSTN^+^* SFs, and *CXCL14^+^* SFs whilst pathological patterns included: fibroid (enriched with SF, leukocyte poor), myeloid (dominated by macrophages), and lymphoid (T and B cell dominant) pathotypes. Disease parameters in fibroid and lymphoid pathotypes appeared to be correlated with presence of *PRG4^+^* and *POSTN^+^* SFs respectively, whilst CXCL14^+^SF showed negative correlation with disease severity in all pathotypes (49, 50).

In the study by Ding et al. integrating eQTL, GWAS, scRNA-seq and bulk RNA-seq multiple datasets and various algorithms, T cell profile and microenvironment in RA were explored to identify T cell-related diagnostic features. For model construction nine algorithms were used including LASSO, XGB, Gradient boosting machine (GBM), Generalized linear model (GLM), Neural network (NNET), SVM, K-nearest neighbours (KNN), Random Forest and Decision tree (DT). Among mentioned above algorithms, five are AI (XGB, GBM, NNET, SVM, KNN). The authors obtained eight T cell-related diagnostic features, which were used for construction of T cell-related diagnostic model (ICOS, IL6ST and PPP1CB were risk factors for RA, whilst GADD45A, CD3D, SLFN5, PIP4K2A and MIER1 were protective factors) (51).

Using the DeepTopics framework Periyakoil et al. identified specific cellular communities (topics) within rheumatoid synovial tissue, with ectopic lymphoid structures (ELS) on histology, that may serve as potential biomarkers of disease activity. Among 22 identified topics across tissue samples, one topic was enriched in IgM^+^ plasma cells and naive B cells (IgM^+^ IgD^+^ TCL1A^+^), particularly in patients with high rheumatoid factor titers, suggesting a link between local B-cell activation and systemic autoantibody production. Another one was characterised by CD34^+^ fibroblast-like synoviocytes and LYVE1^+^ MERTK^+^ macrophages, indicating a distinct stromal-macrophage niche potentially involved in tissue repair or immune regulation. Together, as shown in **Table 2** these AI-defined cellular architectures provide new insight into the heterogeneity of rheumatoid inflammation and its immunopathogenic drivers (52).

Interestingly, a novel ML approach, called Graph-based Gene expression Module Identification (GbGMI) helped to identify factors, beyond inflammation, that relate to and might mediate joint pain in low inflammatory synovium (53). Using sorted-cell subsets and scRNA-seq data, Bai et al. determined that lining fibroblasts express the majority of pain-associated genes, especially those predicted to interact with the dorsal root ganglion of sensory nerves. This led to the discovery that synovial lining fibroblasts produce Netrin-4 (Net4–87 or NTN4), which significantly augments branching of pain-sensitive CGRP^+^ sensory nerves *in vitro.* The fact that synovial fibroblasts can enhance the outgrowth of CGRP^+^ pain-sensitive nerve fibres can explain why RA patients with limited synovial inflammation, also known as “fibroid” or “low inflammatory” synovium, have as much pain as those with extreme inflammation (53).

Although scVI is becoming increasingly popular in the analysis of single-cell RNA-seq data, there are still no published studies using this tool in the field of rheumatology.

## 5 Advantages

The main advantage of AI-based big data analysis in single-cell and spatial transcriptomics in RA is its ability to rapidly and efficiently identify patterns and trends within large datasets, which may serve as potential biomarkers of disease development. Due to the complexity of such data, this type of analysis would not be feasible using conventional human-driven approaches. Moreover, AI-based bulk data analysis can be integrated with single-cell data, resulting in more precise and in-depth insights.

## 6 Limitations

Nowadays, the analysis of medical data requires the use of complex algorithms capable of detecting subtle patterns, reducing dimensionality, and making highly accurate predictions. The most commonly applied approaches include classification and regression techniques, as well as feature selection and gene co-expression network analyses. AI is a powerful tool that is extremely valuable in medicine and science; however, there are areas in which it cannot replace humans or traditional methods, particularly in the context of real-world clinical practice. AI cannot substitute for a deep understanding of biology, clinical responsibility, ethical judgment, laboratory experience, or human interaction — it can only support these elements.

Moreover, public debate requires justification of the high costs associated with AI-based diagnostics in order to legitimise their implementation in routine clinical practice. While AI tools can assist in identifying molecular signatures, caution is necessary, as such signatures may be biologically uninterpretable, unstable, or impossible to validate clinically. This limitation arises from the fact that biological complexity does not scale linearly with the amount of available data.

AI tools usage will probably accelerate the discovery and validation of potential predictive biomarkers that enable personalised treatment strategies. These strategies are better understood as involving a limited number of stratified patient groups rather than fully individualised therapies, due to biological heterogeneity amongst patients, disease dynamics over time, treatment costs, and logistical constraints.

## 7 Conclusions

In the present review, we aim to summarise current knowledge and provide an updated overview of how bulk, single-cell, and spatial transcriptomic approaches, analysed using AI-driven methods, are uncovering disease-specific gene expression programs, pathogenic cell populations, and inflammatory microenvironments that remain beyond the reach of conventional immunological techniques in the investigation of RA pathogenesis.

AI, including ML tools are essential for interpreting these high-dimensional datasets, enabling data integration, molecular disease stratification, biomarker discovery, and prediction of therapeutic responses. These advances support the transition towards precision medicine in rheumatology by identifying biologically and clinically meaningful patient subgroups.

Despite their transformative potential, AI-based approaches cannot replace biological knowledge, clinical expertise, or ethical responsibility. Molecular signatures derived from computational analyses may be difficult to interpret or validate clinically, reflecting the complexity of biological systems. Therefore, careful validation, clinical oversight, and cost-effectiveness considerations are critical for translating these technologies into routine practice.
